# Choice of oncologist and influencing factors: analysis of the Pancreatic Cancer Action Network registry

**DOI:** 10.1093/oncolo/oyaf406

**Published:** 2025-12-13

**Authors:** Rajiv Agarwal, Jennifer G Whisenant, Lili Sun, Fei Ye, Michael B LaPelusa, Lynn M Matrisian, Dana B Cardin, Jordan D Berlin

**Affiliations:** Department of Medicine, Vanderbilt University Medical Center, Nashville, TN 37232, United States; Vanderbilt-Ingram Cancer Center, Nashville, TN 37232, United States; Department of Medicine, Vanderbilt University Medical Center, Nashville, TN 37232, United States; Vanderbilt-Ingram Cancer Center, Nashville, TN 37232, United States; Department of Biostatistics, Vanderbilt University Medical Center, Nashville, TN 37203, United States; Vanderbilt-Ingram Cancer Center, Nashville, TN 37232, United States; Department of Biostatistics, Vanderbilt University Medical Center, Nashville, TN 37203, United States; Department of Medicine, Vanderbilt University Medical Center, Nashville, TN 37232, United States; The Pancreatic Cancer Action Network, El Segundo, CA 90245, United States; Department of Medicine, Vanderbilt University Medical Center, Nashville, TN 37232, United States; Vanderbilt-Ingram Cancer Center, Nashville, TN 37232, United States; Department of Medicine, Vanderbilt University Medical Center, Nashville, TN 37232, United States; Vanderbilt-Ingram Cancer Center, Nashville, TN 37232, United States

**Keywords:** pancreatic cancer, patient-reported outcomes, communication, decision-making, patient-oncologist relationship, second opinion

## Abstract

Choice of oncologist by patients with pancreatic cancer is a complex personal decision. We conducted a retrospective analysis of the Pancreatic Cancer Action Network registry to explore whether age, gender, disease status, feelings of trust and comfort, and the opportunity for clinical trial participation influenced patient choice. Of 110 participants who completed the “Information about Choosing an Oncologist Survey,” 68 (61.8%) reported visiting another oncologist. Feeling comfortable and trusting their first oncologist decreased the likelihood of seeking a second opinion (OR: 0.08; CI: 0.01-0.42; *P* = .005). Patients with resectable disease were also less likely to visit another oncologist compared to patients with borderline resectable or locally advanced disease (OR: 0.28; CI: 0.08-0.95; *P* = .042). Age, gender, and the opportunity for clinical trial participation did not influence patient choice. Most patients who saw additional oncologists did so because of recommendations from friends or family members. This analysis leveraged patient-reported outcomes to highlight determinants of patient decision-making.

## Introduction

Pancreatic cancer is a highly lethal disease^1^ that is associated with significant symptom burden and treatment-related toxicity. At the time of diagnosis, patients with pancreatic cancer may have multiple factors that influence how they choose their care. General determinants of patient choice of healthcare providers have been previously identified, and include various degrees of importance assigned to patient and provider characteristics, factors such as accessibility and cost of care, and clinical outcomes.^2^ While several studies have examined how patients choose surgeons and primary care physicians, less is known on how patients with cancer choose their oncologists.^3–5^

Patient-reported outcomes (PROs) afford the opportunity to better understand influential factors that guide patient decision-making and choice. A limited number of studies use online PRO registries from patients and caregivers for a single disease process. The Pancreatic Cancer Action Network (PanCAN) patient registry is an online, global database specific to pancreatic cancer that enables patients to self-report sociodemographic information, disease and management characteristics, and PROs. The development of the database and questionnaires, along with the patient experience, has been described previously.^6–8^ The first publication by Gupta et al.^6^ demonstrated the feasibility, usability, and potential research opportunities of a PRO registry that includes 27 unique surveys that ask patients to report on various topics about their health, disease, and cancer treatment (see online Supplementary Material). The second publication by McNearny et al.^7^ reported results of pre-diagnosis pancreatic cancer pain and its association with post-diagnosis symptom burden, metastatic disease, and increased resource utilization. More recently, a quantitative analysis has been published to better understand and describe the experience of pain and depression reported by patients with pancreatic cancer.^8^ We utilized the real-world data from this registry to investigate specific factors that were most important to a patient when choosing their oncologist.

## Methods

**Figure 1.**
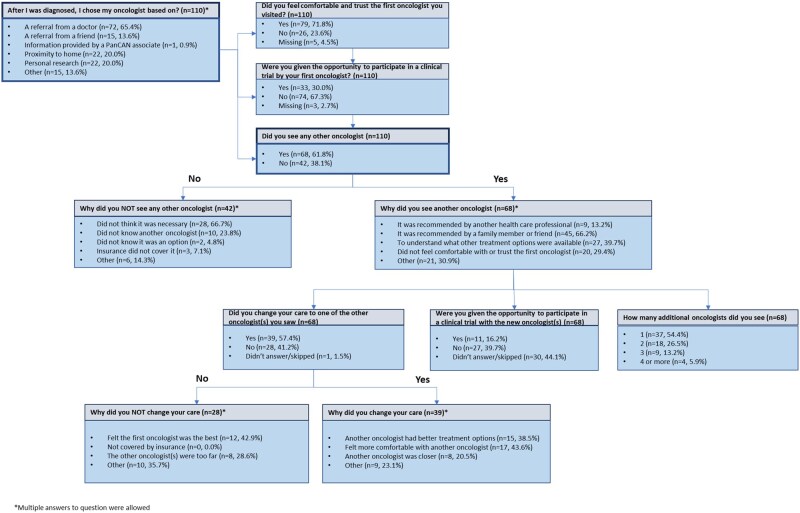
“Information about Choosing an Oncologist” Survey. Survey questions and answers with associated branching logic are presented for the “Information about Choosing an Oncologist Survey” within the PanCAN PRO database. Numbers and percentages for each question and answer are included.

This retrospective study analyzed data submitted in the “Information about Choosing an Oncologist Survey,” which asks questions related to reasons that influence a patient’s choice of oncologist (Figure 1). The main outcome measure was whether a patient visited a second oncologist. Potential influencing factors were selected based on review of survey questions and clinical reasoning. Race was not included in the analysis, as most patients who completed the “Information about Choosing an Oncologist Survey” were white. Demographic characteristics were compared between participant groups (“No” did not see another oncologist vs “Yes” did see another oncologist) using appropriate Chi-square tests and *t*-tests, with adjusted *P*-values generated using the False Discovery Rate method for multiplicity (Table 1). Logistic regression models were used to estimate effects of availability of clinical trial opportunity and the patient’s trust and comfort with their first oncologist (yes/no) on whether a patient met with other oncologist (yes/no), adjusted for age, gender, and disease status. Due to the small number of missing covariate data, multiple imputation analysis was not reported. All statistical analyses were conducted in R 4.3.0.

**Table 1.** Participant demographics.

Variables of interest	Total (*n* = 110)	Did the participant see another oncologist?		*P*-Value (adjusted *P*-value)
		No (*n* = 42)	Yes (*n* = 68)	
**Median age, years (Range)**	63.8 (21.9,86.3)	65.9 (37.9, 77.5)	62.3 (21.9, 86.3)	.676 (1)
**Gender**				1 (1)
** Female**	41 (37.3%)	16 (38.1%)	25 (36.8%)	
** Male**	58 (52.7%)	22 (52.4%)	36 (52.9%)	
** Missing**	11 (10.0%)	4 (9.5%)	7 (10.3%)	
**Race/ethnicity**				.266 (1)
** Asian**	5 (4.5%)	4 (9.5%)	1 (1.5%)	
** Hispanic/Latinx**	5 (4.5%)	2 (4.8%)	3 (4.4%)	
** White**	88 (80.0%)	32 (76.2%)	56 (82.4%)	
** Missing**	12 (10.9%)	4 (9.5%)	8 (11.8%)	
**Current disease status**				.756 (1)
** Borderline resectable**	28 (25.5%)	10 (23.8%)	18 (26.5%)	
** Locally advanced**	21 (19.1%)	6 (14.3%)	15 (22.1%)	
** Metastatic**	29 (26.4%)	11 (26.2%)	18 (26.5%)	
** Resectable**	30 (27.3%)	13 (31.0%)	17 (25.0%)	
** Missing**	2 (1.8%)	2 (4.8%)	0 (0%)	
**Did you feel comfortable with and trust the first oncologist you saw?**				.003 (0.017)
** No**	26 (23.6%)	3 (7.1%)	23 (33.8%)	
** Yes**	79 (71.8%)	37 (88.1%)	42 (61.8%)	
** Missing**	5 (4.5%)	2 (4.8%)	3 (4.4%)	
**Were you given the opportunity to participate in a clinical trial by this oncologist?**				.944 (1)
** No**	74 (67.3%)	27 (64.3%)	47 (69.1%)	
** Yes**	33 (30.0%)	13 (31.0%)	20 (29.4%)	
** Missing**	3 (2.7%)	2 (4.8%)	1 (1.5%)	

Demographic information, responses to if a patient trusted their first oncologist, and responses to if they were offered a clinical trial for all participants and stratified by whether participants met with another oncologist. *P*-values were calculated between groups using Chi-square tests and *t*-tests for categorical and numerical variables, respectively. Adjusted *P*-values were also calculated using the false discovery rate (FDR) method for multiplicity.

## Results

As of December 2020, 2187 participants visited the registry and completed at least one survey. Of these, 110 participants completed the “Information about Choosing an Oncologist Survey” and were included in this analysis (median age 63.8 years [range: 21.9-86.3], 37.3% women, 80.0% White). Figure 1 presents the survey questions and potential answers used in this analysis with the associated branching logic. Demographic information and responses to if a patient trusted their first oncologist and if they were offered a clinical trial are summarized in Table 1 for all participants, as well as stratified by whether those participants met with another oncologist.

For the primary endpoint, 42 (38.2%) and 68 (61.8%) participants answered “No” or “Yes”, respectively, to if they visited another oncologist. Feeling comfortable and trusting the first oncologist significantly decreased the likelihood of seeking a second opinion (OR: 0.08; CI: 0.01-0.42; *P* = .005). Patients with resectable disease were also less likely to visit another oncologist compared to patients with borderline resectable or locally advanced disease (OR: 0.28; CI: 0.08-0.95; *P* = .042). Age (OR: 0.94; CI: 0.51-1.75; *P* = .856), gender (OR: 0.88; CI: 0.32-2.38; *P* = .799), and the opportunity to participate in a clinical trial (OR: 0.68; CI: 0.25-1.89; *P* = .462) did not influence the decision to visit another oncologist.

Patient-reported reasons for why they chose to visit with another oncologist and why they changed their care are further described in Figure 1. Most patients chose their oncologist based on physician referrals, and of those who did not seek a second opinion, 66.7% (28/42) felt it was not necessary. Interestingly, of the 68 respondents that visited with additional oncologists, 45 (66.2%) did so because of recommendations from family members or friends, and 31 (45.6%) saw more than one additional oncologist. The majority (42.9%; 12/28) of patients who did not change their care after visiting with another oncologist reported that their first oncologist was the best. Of note, most patients who changed their care did so because they felt more comfortable with their new oncologist (43.6%; 17/39).

## Discussion

Choice of oncologist by patients with pancreatic cancer is a complex personal decision, requiring patients to consider multiple factors.^9^ This study leveraged information and patient-reported outcomes from the PanCAN global registry to evaluate some of the determinants of patient choice. Our analysis demonstrates that clinical disease status (resectable vs borderline/locally advanced unresectable disease) and patient-reported trust and comfort were both influencing factors, whereas the opportunity to participate in a clinical trial did not affect patient choice.

It is not surprising that disease status influences choice of oncologist, as patients with pancreatic cancer are understandably hopeful for curative intent treatment. With resectable disease, there is more certainty regarding management and the potential for surgery; thus, these patients may have fewer reasons to seek additional medical opinions. However, patients who have borderline resectable or locally advanced unresectable disease often are informed that surgery may not be guaranteed and its possibility will depend on tumor response to neoadjuvant treatment. This uncertainty may lead patients and caregivers to meet with additional oncologists, either for confirmation or for pursuing different recommendations. In fact, nearly 40% of the patients in our analysis who visited with another oncologist did so to learn about other treatment options.

Perhaps the most notable finding of this study is that patient-reported feelings of trust and comfort with their first oncologist impact whether patients sought a second opinion and ultimately changed oncologists for the management of their disease. Our analysis shows that having trust and comfort reduced the odds of wanting additional recommendations. Moreover, though only 30% of patients who visited with additional oncologists reported doing so due to a lack of trust or comfort with their first oncologist, most who elected to change their care reported that comfort with their new provider influenced their choice. These findings are consistent with existing data that demonstrate that reduced trust can motivate patients with cancer to seek a second opinion.^10^ Qualitative analyses have additionally shown that physician attributes, including their care, curiosity, and open-mindedness, are important for trustful relationship building.^11^ Finally, the dynamics of trust development in the context of a clinical encounter in oncology are contingent on multiple patient and provider factors, including care continuity and communication.^12^ Therefore, while trust and comfort can be established with time, it can also be fostered by effective communication and building rapport with transparency and empathy.

As expected, friends and family members may also influence patient decision-making, with nearly 2/3 of patients reporting that they met with additional oncologists because of recommendations from loved ones. When possible, oncologists should include patients’ friends or family members in conversations about pancreatic cancer to address their specific concerns or questions, ensure that information is conveyed in ways that are understood, and strengthen the therapeutic alliance.

Interestingly, the opportunity to participate in a clinical trial did not impact choice of oncologist. This result does not negate the importance of clinical trials and the potential impact they make on survival outcomes for patients with pancreatic cancer, but instead highlights that the prospect of clinical trial participation does not independently influence patient choice. One possible explanation for this finding is that whether a clinical trial is being offered is not as important as how oncologists make patients feel heard and discuss available treatment options (including standard of care or investigational treatment) to form a patient-centered management plan that accounts for patients’ preferences and values.^13^

Our analysis has limitations. Race/ethnicity was not included as a covariate in the regression model due to limited diversity in our cohort, and therefore we were unable to assess if racial/ethnic disparities impact patient choice of oncologist. Another limitation of the study is the smaller number of participants within the PanCAN registry who completed the “Information about Choosing an Oncologist Survey.” While PROs enable us to examine the patient experience and what shapes patients’ choices,^13^^,^^14^ survey-based studies still rely on voluntary participation and are susceptible to missing data. Moreover, type of insurance, potential financial toxicity, oncology practice setting (academic vs community), distance and type of residence (urban vs rural), and geographical location of survey respondents are additional factors that could have impacted the choice a patient makes when seeking a second opinion. In our cohort, only three respondents (7.1%) listed insurance as a barrier to visiting with another oncologist. In addition, only 20% of respondents indicated that they chose their oncologist due to proximity to home. With that said, our findings from the PanCAN registry may not be generalizable to the global pancreatic cancer population and further investigation is needed to evaluate the impact of these additional variables on patient choice. It is also important to note that patients who join the PanCAN registry may be more proactive in reporting their cancer experience, and as a result, the results of this study may have differed if conducted by an institution or cooperative group. Therefore, evaluating factors that influence choice of oncologist in a multi-institutional or cooperative group study would help provide external validation of our results. Lastly, we are unable to discern from the survey data what contributes to feelings of trust and comfort (eg, provider communication styles, oncologists’ ratings, provision of early supportive care, or reputation of cancer hospitals). Further research is needed to evaluate how oncologists learn and develop skills to build connection with their patients, with particular attention to improving patient-centered communication.

## Conclusion

In summary, our study demonstrates that resectable disease status and engendering trust and comfort are two impactful factors for patients with pancreatic cancer when choosing their oncologist. This is one of few studies that utilizes information and PROs from a global registry to better understand what influences patient decision-making. Future studies should prospectively explore what factors lead to patient-reported feelings of trust and comfort with oncology teams, measure trust quantitatively using validated instruments,^15–18^ and importantly determine whether early supportive care integration to address the high physical and emotional needs of patients with pancreas cancer can nurture trust in the therapeutic alliance.^7^^,^^19^^,^^20^

## Supplementary Material

oyaf406_Supplementary_Data

## Data Availability

The data underlying this article were provided by The Pancreatic Cancer Action Network (PanCAN) by permission. De-identified data and data dictionary will be shared on request to the corresponding author with permission and data use agreement with PanCAN.

## Supplementary material

Supplementary material is available at *The Oncologist* online. List of all surveys available to registry users: Unique surveys within the PanCAN registry that ask patients to report on various topics pertaining to their health, care choices, cancer treatment, and cancer-related symptoms.
