# Supplementary material for: Choice of oncologist and influencing factors: analysis of the Pancreatic Cancer Action Network registry
Source: Oncologist. 2025 Dec 13;31(1):oyaf406. doi: 10.1093/oncolo/oyaf406 (PMC12782827; doi:10.1093/oncolo/oyaf406)
Supplement: oyaf406_Supplementary_Data [file oyaf406_supplementary_data.docx]

**Supplemental Material: List of all surveys available to registry users.**

| **General Information** | |
| --- | --- |
|  | Pancreatic Cancer Experience Basics  Health Assessment  General Demographic Information |
| **General Medical Health** | |
|  | Diabetes  Family History  Other Cancers  Tobacco Use |
| **Care Choices** | |
|  | Clinical Trial  Information About Choosing an Oncologist |
| **Diagnostics** | |
|  | CA19.9  Diagnosis and Procedures  Know Your Tumor Follow Up  Molecular Profiling  Scans  Test Results  Detailed Diagnosis |
| **Cancer Treatment** | |
|  | Drug Therapy  Radiation  Surgery |
| **Symptoms/Management** | |
|  | Depression Management  Digestion  Fatigue Management  Nausea Management  Pain Management  Pancreatic Enzymes |
